# Salivary peptest for laryngopharyngeal reflux and gastroesophageal reflux disease: A systemic review and meta-analysis

**DOI:** 10.1097/MD.0000000000026756

**Published:** 2021-08-13

**Authors:** Zihao Guo, Jiali Jiang, Hao Wu, Jinxia Zhu, Shutian Zhang, Chuan Zhang

**Affiliations:** a Department of Gastroenterology, Beijing Tong Ren Hospital, Capital Medical University, Beijing, China.; b Department of Cardiovascular diseases, Beijing Tong Ren Hospital, Capital Medical University, Beijing, China.; c Department of Physiology and Pathophysiology, School of Basic Medical Sciences, Capital Medical University, Beijing, China.; d Department of Gastroenterology, Beijing Friendship Hospital, Capital Medical University, Beijing, China.; e National Clinical Research Center for Digestive Disease, Beijing Digestive Disease Center, Beijing, China.; f Beijing Key Laboratory for Precancerous Lesion of Digestive Disease, Beijing, China.

**Keywords:** diagnosis, gastroesophageal reflux disease, laryngopharyngeal reflux, meta-analysis, pepsin, Peptest, salivary

## Abstract

**Background:**

A rapid lateral flow test (Peptest) to detect pepsin in saliva/sputum has been considered as a valuable method for diagnosing laryngopharyngeal reflux (LPR) and gastroesophageal reflux disease (GERD). The aim of this meta-analysis is to analyze the utility of Peptest for diagnosis of LPR and GERD.

**Methods:**

PubMed, EMBASE, and the Cochran Library (from January 1980 to 26 January 2020) were searched for pepsin in saliva for LPR/GERD diagnosis. Sensitivity, specificity, positive likelihood ratio, negative likelihood ratio, diagnostic odds ratio, and area under the curve data were summarized to examine the accuracy.

**Results:**

A total of 16 articles that included 2401 patients and 897 controls were analyzed. The pooled sensitivity and specificity for the diagnosis of GERD/LPR with Peptest were 62% (95% confidence interval [CI] 49%–73%) and 74% (95% CI 50%–90%), respectively. The summarized diagnostic odds ratio and area under the curve were 5.0 (95% CI 2–19) and 0.70 (95% CI 0.66–0.74), respectively.

**Conclusion:**

Peptest shows moderate diagnostic value for LPR and GERD. More studies with standard protocols should be done to verify its usefulness.

## 1 Introduction

The backflow of stomach contents can cause a series of problems. Gastroesophageal reflux disease (GERD) is defined as a disorder that develops when the stomach contents backflow into esophagus causes symptoms, presenting as heartburn and acid regurgitation. When the stomach contents backflow into supra-esophageal, it causes extraesophageal symptoms, such as laryngopharyngeal reflux disease (LPRD), which is defined as the backflow of gastric or gastroduodenal contents into the laryngopharynx. GERD is a common disease affecting about 10% to 40% of the Western adult population and 17% of the Asian adult population.^[1–3]^ Approximately 50% of patients with LPRD are affected in the voice center and account for consultation of about 10% of outpatients of the ear, nose, and throat department.^[2,3]^ LPRD not only causes annoying symptoms such as hoarseness, sore throat, odynophagia, globus sensation, and throat clearing, but also may be related to reflux laryngitis, reflux asthma, dental erosion, pharyngitis, sinusitis, idiopathic lung fibrosis, and even laryngeal malignancy.^[4–6]^

The diagnosis of GERD/LPRD is initially based on the symptoms and endoscopic findings of the patients. The questionnaires for GERD/LPRD are lack of sensitivity and specificity. For GERD, the reflux disease questionnaire and gastroesophageal reflux disease questionnaire are mostly used, while the Reflux Symptom Index is mostly used for LPRD.^[7,8]^ Erosive esophagitis is found in only 30% of patients with GERD and low-grade esophagitis could be found in asymptomatic controls.^[9,10]^ The most used score system for LPRD of fiberoptic laryngoscopy is the Reflux Finding Score. However, a literature review showed that the laryngopharyngeal mucosal signs associated with laryngopharyngeal reflux (LPR) were also shown in healthy volunteers.^[11,12]^ Multichannel intraluminal impedance (MII) combined with pH monitoring can provide more comprehensive data and clues for the reflux events.^[13]^ However, the procedure of pH/pH-MII monitoring is invasive and expensive.^[14,15]^

**Figure 1:**
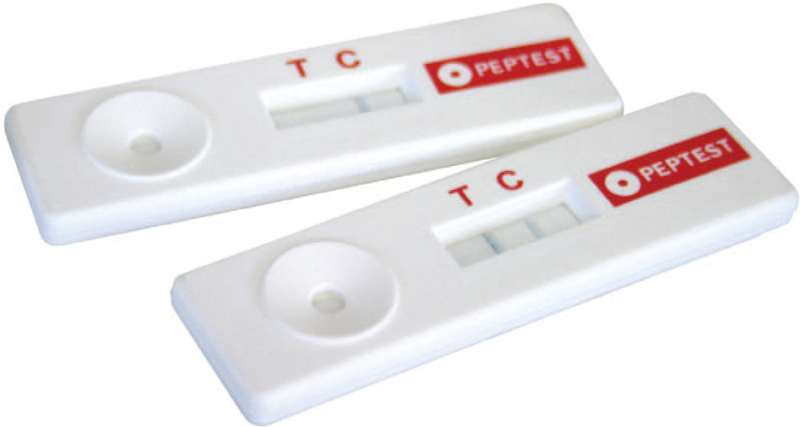
Peptest strip is used to assess for the presence of pepsin in saliva. Reprinted with permission from https://www.peptest.co.uk/peptest/. ^©^ Peptest, United Kingdom. All permission requests for this image should be made to the copyright holder.

Pepsin is only produced in the stomach, so it is a specific biomarker for gastric reflux and can be detected in all kinds of reflux contents, such as saliva, sputum, secretory otitis media, and even in tears.^[16,17]^ Detection of pepsin in saliva is considered as a noninvasive and convenient diagnostic method for LPRD/GERD. The fibrinogen digestion was the first method to be used for pepsin detection.^[18]^ In addition, western blot and enzyme-linked immunosorbent assay have also been used for pepsin assay.^[19–21]^ However, these methods are both time consuming and laborious. A rapid lateral flow test (Peptest, RD Biomed Limited, UK, as shown in Fig. 1) to detect pepsin in saliva/sputum has shortened the salivary pepsin assay to several minutes and offered a strong predictive value for LRPD/GERD diagnosis.^[22]^ There are several studies evaluating the diagnostic value of Peptest for LRPD/GERD, but they showed different results.^[19,22,28–41]^

In the present research, we did a meta-analysis and systematically reviewed the potential of Peptest as a diagnostic tool of LPRD/GERD.

## 2 Materials and methods

### 2.1 Search strategy and study inclusion

This study was performed in accordance with the guidelines for the Preferred Reporting Items for Systematic reviews and Meta-Analyses.^[23]^ Two investigators (Guo and Jiang) independently searched the published articles in multiple databases, including PubMed, EMBASE, and the Cochran Library (from January 1980 to 26 January 2020). The search strategies in PubMed and EMBASE were “Search ((saliva[Title/Abstract]) OR salivary[Title/Abstract]) OR spit[Title/Abstract])) OR ‘Saliva’[Mesh])) AND ((pepsin[Title/Abstract]) OR peptest[Title/Abstract]) and (‘saliva’/exp OR ‘saliva’ OR ‘salivary’:ab,ti OR ‘spit’:ab,ti) AND (‘pepsin’:ab,ti OR ‘peptest’:ab,ti),” respectively. References of review articles and previously published meta-analyses were also searched manually. For the reason that this article is a meta-analysis, the study does not need an ethics committee or institutional review board approval.

### 2.2 Inclusion and exclusion criteria

Only studies that met the following criteria were included:

(1) just for the adult population,
(2) assessed the accuracy of saliva pepsin for LPR or GERD,
(3) provided sufficient information to construct the 2 × 2 contingency table.

We only included original articles written in English. Animal experiments, correspondences, reviews, case reports, conference abstract, and editorials were excluded.

### 2.3 Procedures

Two investigators (GZH and JJL) extracted data from the enrolled studies and assessed the methodological quality independently. The extracted data included year, methodology characteristics, country of origin, details of the pepsin assays, and cut-off value. The methodological quality of the studies was assessed with the Quality Assessment of Diagnostic Accuracy Studies-2 (QUADAS-2). The QUADSA-2 has been used in systematic reviews to evaluate the methodological quality of studies. The QUADAS-2 consists of 4 domains in terms of risk of bias: patient selection, reference standard, index test, and flow and timing, and the first 3 domains, including patient selection, index test, reference standard, are also evaluated in terms of concerns of the applicability. The risk of bias for each domain is rated as high, low, or unclear according to answers of signalling questions. The study is judged as “low risk of bias” or “low concern regarding applicability” when all domains relating to bias or applicability were “low.” In contrast, the study is rated “at risk of bias” or “concerns regarding applicability” when one or more domains were judged as “high” or “unclear.”^[24]^ Discrepancies were resolved in a consensus meeting or by referral to a third investigator (WH).

### 2.4 Statistical analysis

All the extracted data were imported into STATA ver. 13.0 (Stata Corp, College Station, TX) and Review Manager (version 5.2 of Windows; Cochrane Collaboration, Oxford, UK). The sensitivity, specificity, pooled positive likelihood ratio, pooled negative likelihood ratio, diagnostic odds ratio, and corresponding 95% confidence intervals (CIs) were calculated from true positive, false positive, false negative, and true negative, which were extracted from each study before data pooling. The summary receiver operator characteristic curve was used to evaluate the overall performance of saliva pepsin in LRP/GERD patients. A 95% prediction region was used to predict the sensitivity and specificity of a future study to lie.^[25]^ The *I*^2^ statistic was done to assess the statistical heterogeneity. *P* > .10 indicated no significant heterogeneity for the *Q* statistic, while *P* ≤ .10 indicated significant heterogeneity. If the heterogeneity was high (*P* < .05, *I*^2^ > 50%), a random effect model was used. Meta-regression analyses were conducted on the basis of cut-off value and diagnostic criteria.^[26]^ Potential publication bias was evaluated by Deeks’ asymmetry test.^[27]^

## 3 Results

### 3.1 Study characteristics and quality assessment

**Figure 2:**
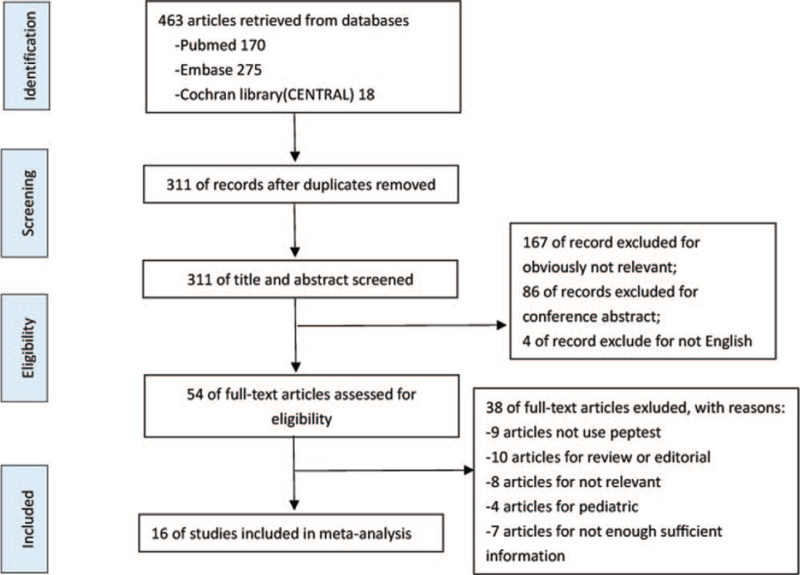
PRISMA flowchart to obtain study data for meta-analysis. PRISMA, Preferred Reporting Items for Systematic reviews and Meta-Analyses.

Detailed search steps were described in Figure 2. A total of 463 potentially relevant articles were identified for retrieval. There were 311 of records left after duplicates were removed. After screening the titles and abstracts, 167 irrelevant articles showing contents, such as “saliva in esophageal defense,” “development of the human gastrointestinal tract,” and “submandibular salivary proteases” were excluded from the list, and as the contents such as 86 articles for conference abstracts were excluded, and 4 articles were not written in English. Searching for the reference lists of the identified articles and previous systematic reviews did not identify any more relevant articles.^[42–45]^ After detailed inspection of 54 full articles, 38 of full-text articles were further excluded due to the following factors: no Peptest method used, review or editorial, irrelevant, pediatric, or no sufficient information obtained. Sixteen articles ultimately met our predefined inclusion criteria and were included in the final analysis.^[19,22,28–41]^ Details of these studies were shown in Table 1.

**Table 1:** Characteristics of studies included in the meta-analysis.

No.	Author	Year	Country	Sample size	Diagnostic criteria	Cut-off (ng/ml)	TP	FP	TN	FN	Sensitivity %	Specificity %
1	Matsumura^[28]^	2020	Japan	38GERD/64SC	24 h pH-MII	187	19	36	28	19	50.0	44.2
2	Weitzendorfer^[29]^	2019	Australia	41LPR/29SC	24 h pH-MII	216	17	4	25	24	41.5	86.2
3	Dettmar^[30]^	2019	UK	985LPR/22HC	Not clear	16	732	0	22	253	76.4	100
4	Bor^[31]^	2019	UK	14LPR/6SC	24 h pH-MII	16	11	5	1	3	78.5	16.7
5	Race^[32]^	2019	UK	30GERD/20HC	24 h pH-monitoring	16	25	15	5	5	83.3	25
6	Wang^[33]^	2019	China	709GERD/323HC	RDQ	75	603	130	194	106	85.0	60.0
7	Woodland^[34]^	2018	UK	24GERD/20SC	24 h pH-MII	210	9	8	15	12	42.9	65.2
8	Dolina^[35]^	2018	Czech	32GERD/11 HC	24 h pH-MII	36	7	5	8	12	36.8	61.5
9	Lleo^[36]^	2018	Spain	180LPR/41SC	RSI	16 (fasting)	72	1	40	108	40.0	97.6
10	Du^[37]^	2017	China	122GERD/128 SC	24 h pH-MII	76	89	15	113	33	73.0	88.3
11	Yadlapati^[38]^	2016	USA	14LPR/18HC	GERDQ and RSI	16	9	8	6	9	50.0	42.9
12	Hayat^[39]^	2015	UK	111 GERD symptoms/100HC	24 h pH-MII	16	66	40	74	18	78.6	64.9
13	Ocak^[40]^	2015	Turkey	18LPR/2SC	24 h 2 channels pH-monitoring	16	6	0	2	12	33.3	100
14	Spyrodoulias^[41]^	2015	UK	40LPR/38SC	RFS	25	31	18	20	9	77.5	52.6
15	Hayat^[19]^	2014	UK	21LPR/10SC	RSI	25	13	4	6	8	61.9	60.0
16	Yuksel^[22]^	2012	UK	22GERD/25SC	48 h-pH monitoring	16	11	2	23	11	50.0	92.0

**Figure 3:**
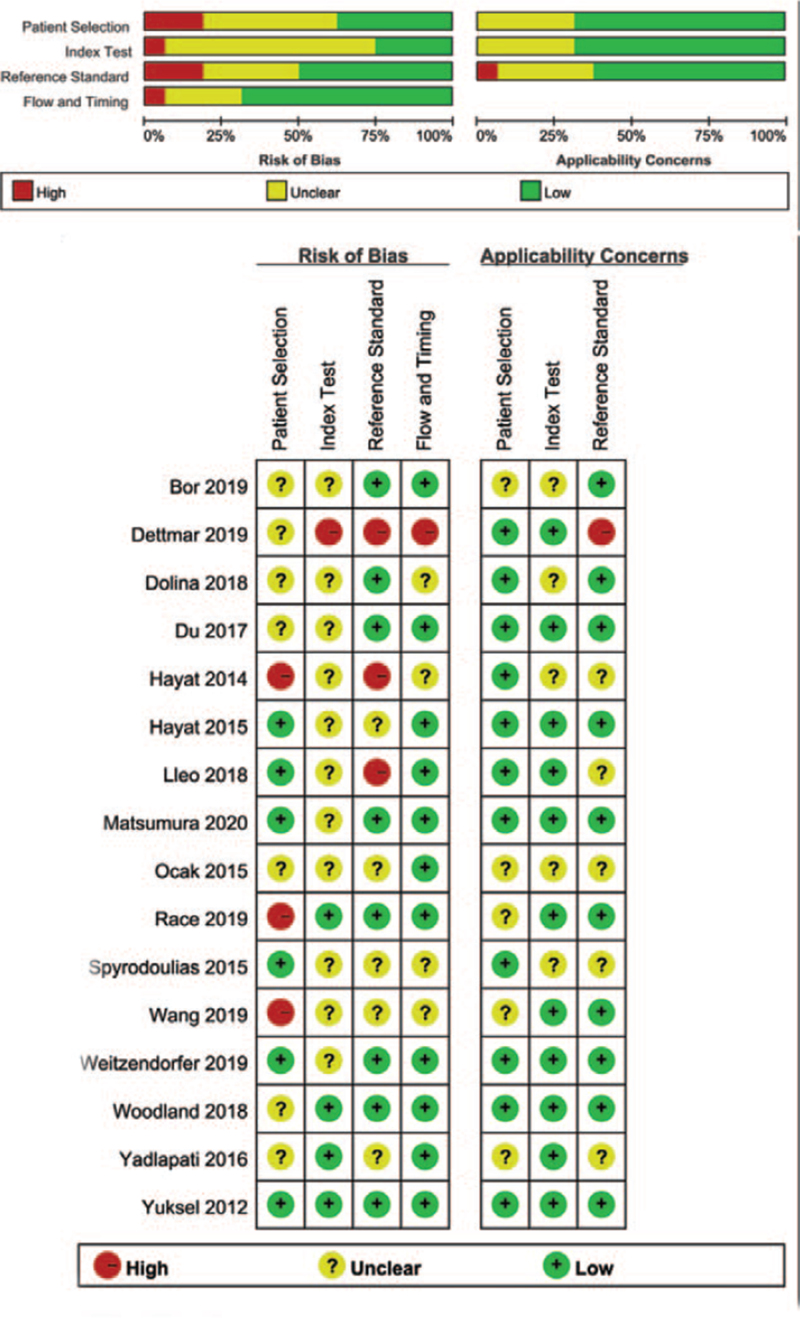
Quality of the studies as assessed by QUADAS questionnaire. Green indicated absence of bias, red indicated the presence of bias, and yellow indicated unclear. QUADAS-2, Quality Assessment of Diagnostic Accuracy Studies-2.

The quality of all included articles was assessed by QUADAS-2 checklist and the summary diagram was shown in Figure 3. Because some studies could not avoid a case–control design,^[19,30–35,37,38,40]^ or the threshold for Peptest was not prespecified,^[19,28,30,31,33–41]^ pH monitoring was not used as the reference standard,^[19,30,33,36,38–41]^ or a lack of information provided with respect to interval between Peptest and reference standard,^[19,30,33,35,41]^ studies were regarded as at risk of bias. In relation to concerns about applicability, 6 studies^[22,28,29,34,37,39]^ were rated as low, other studies^[19,30–33,35,36,38,40,41]^ were regarded having concerns regarding applicability.

### 3.2 Meta-analysis results for diagnostic value

**Figure 4:**
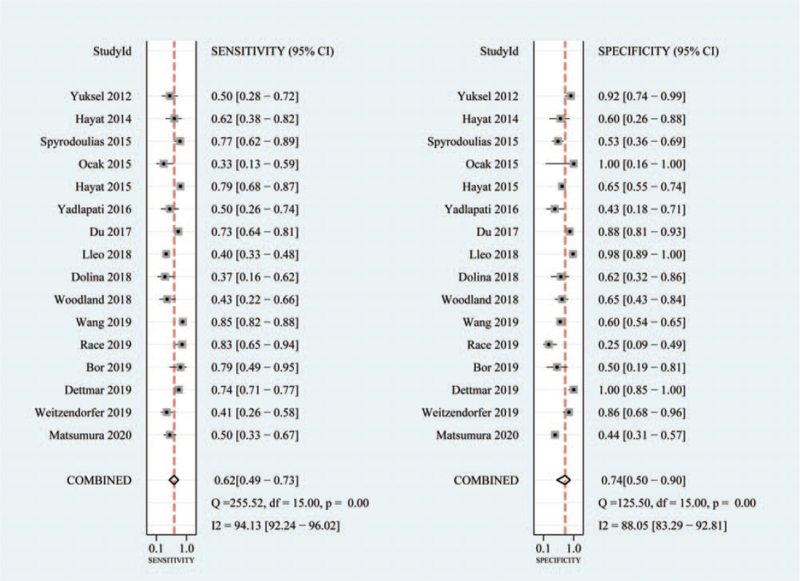
Sensitivity and specificity of salivary Peptest for diagnosis of LPR/GERD. GERD, gastroesophageal reflux disease; LPR, laryngopharyngeal reflux.

**Figure 5:**
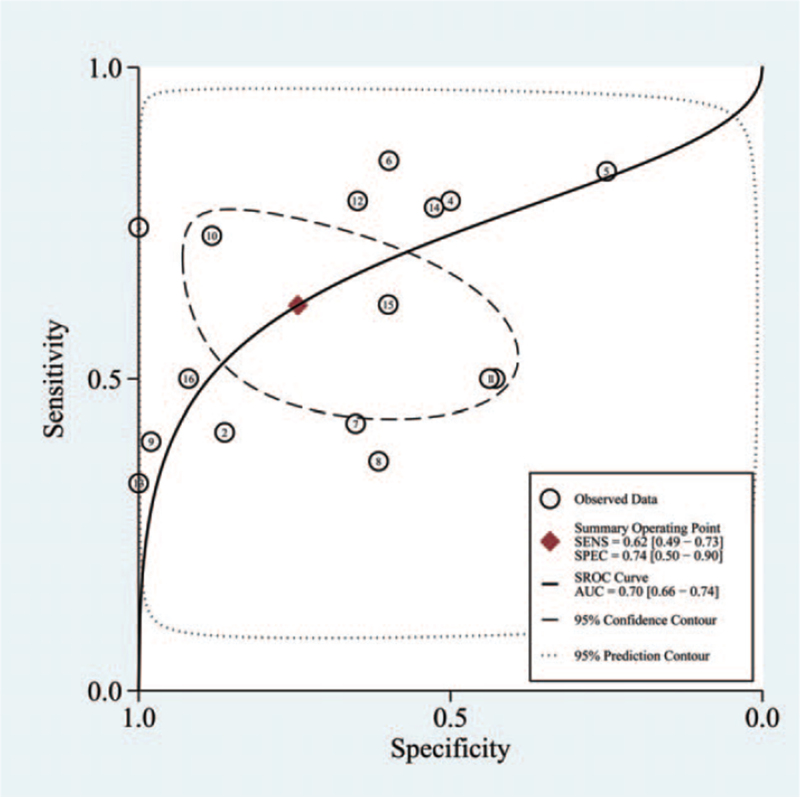
Summary receiver operating characteristic curve of salivary Peptest for diagnosis of LPR/GERD. Each circle showed individual study estimates; inner ellipse represents 95% confidence region, and outer ellipse represents 95% prediction region for a future study. GERD, gastroesophageal reflux disease; LPR, laryngopharyngeal reflux.

A total of 16 articles that included 2401 patients and 897 controls were analyzed for the diagnostic accuracy of salivary Peptest for GERD/LPRD. As shown in Figure 4, pooled sensitivity and specificity were 0.62 (95% CI 0.49–0.73) and 0.74 (95% CI 0.50–0.90), respectively. The pooled positive likelihood ratio and pooled negative likelihood ratio were 2.4 (95% CI 1.0–6.1) and 0.51 (95% CI 0.31–0.86), respectively. The diagnostic odds ratio was 5.0 (95% CI 2–19). The area under the receiver operating characteristic curve was 0.70 (95% CI 0.66–0.74; Fig. 5).

### 3.3 Heterogeneity, subgroup analysis and publication bias

**Figure 6:**
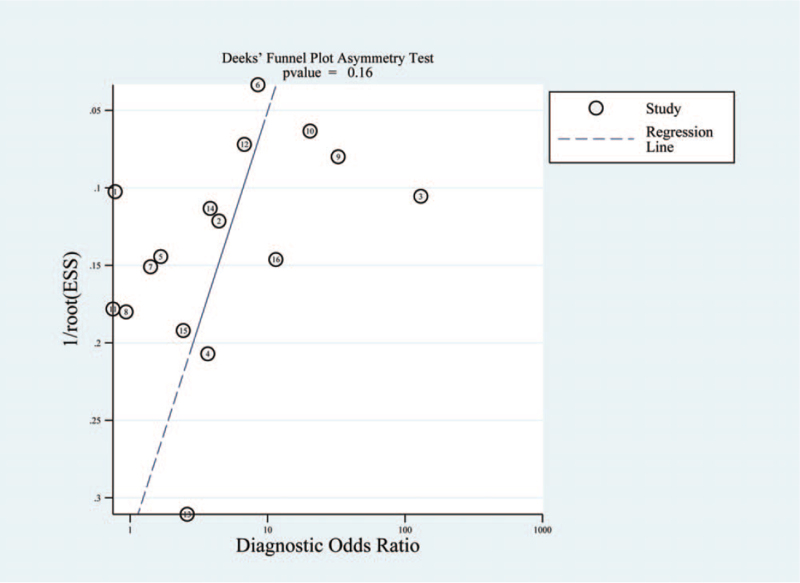
Deeks’ plot of salivary Peptest for diagnosis of LPR/GERD. The plots were in symmetrical funnel shape and *P* values for the Deeks funnel plot asymmetry test were 0.16, indicating that the publication bias was likely absent. GERD, gastroesophageal reflux disease; LPR, laryngopharyngeal reflux.

The proportion of heterogeneity was high (sensitivity: *I*^2^ = 94.13%, *P* = .00; specificity: *I*^2^ = 88.05%, *P* = .00). We did meta-regression analyses to identify the source of heterogeneity. As shown in Table 2, there was low heterogeneity when subgroup analyses were conducted according to cut-off value (>25 ng/ml or ≤ ng/ml) (*I*^2^ = 27%, *P* = .25), region (Western country or Asia) (*I*^2^ = 0%, *P* = .98), sample size (≥50 vs <50) (*I*^2^ = 0%, *P* = .71), diagnosis (GERD or LPR) (*I*^2^ = 55%, *P* = .11), diagnostic method (24 hours pH/pH-MII monitoring or other) (*I*^2^ = 56%, *P* = .10), and control group (health control or symptomatic control) (*I*^2^ = 71%, *P* = .10). The Deeks’ asymmetry test showed no evidence of publication bias (*P* = .16; Fig. 6).

**Table 2:** Subgroup analysis of Peptest for GERD/LPR diagnosis.

Subgroup (number of studies)	Sensitivity	*P*	Specificity	*P*
Region		.71		.97
Western country (n = 12)	0.62 [0.48–0.77]		0.76 [0.53–0.98]	
Asia (n = 4)	0.60 [0.35–0.86]		0.70 [0.26–1.00]	
Sample size		.79		.55
≥50 (n = 9)	0.66 [0.51–0.81]		0.79 [0.55–1.00]	
<50 (n = 7)	0.55 [0.35–0.75]		0.68 [0.33–1.00]	
Cut-off value		.10		.43
>25ng/ml (n = 6)	0.49 [0.29–0.68]		0.62 [0.23–1.00]	
≤25 ng/ml (n = 10)	0.70 [0.56–0.83]		0.81 [0.59–1.00]	
Diagnosis		.09		.04
GERD (n = 8)	0.52 [0.35–0.69]		0.53 [0.21–0.85]	
LPR (n = 8)	0.71 [0.56–0.86]		0.89 [0.74–1.00]	
Diagnostic method		.02		.30
pH/pH-MII (n = 10)	0.51 [0.37–0.66]		0.65 [0.36–0.94]	
Other (n = 6)	0.76 [0.63–0.89]		0.86 [0.65–1.00]	
Control group		.02		.67
Health control (n = 6)	0.76 [0.62–0.89]		0.71 [0.35–1.00]	
Symptomatic control (n = 10)	0.52 [0.37–0.66]		0.77 [0.53–1.00]	

## 4 Discussion

Pepsin is an active enzyme derived from pepsinogen by the action of hydrochloric acid in stomach, so its presence in saliva can only be explained by an episode of reflux. Over the past years, a growing number of studies assessed the salivary pepsin for diagnosis of GERD/LPRD. Multiple methods were used to detect the pepsin in saliva, such as Peptest, western blot, and enzyme-linked immunosorbent assay, among which the Peptest is the most convenient method, and Peptest can shorten the salivary pepsin assay to several minutes.^[42]^ Wang et al did a meta-analysis to assess the diagnostic value of pepsin in saliva for LPR/GERD, which included 11 studies of all kinds of assay type.^[43]^ The results showed that pepsin in saliva has moderate value for LPR/GERD diagnosis, and the area under the curve was 0.71 (95% CI 0.67–0.75). Our study exclusively focused on the diagnostic value of Peptest on LPR/GERD. In this meta-analysis, the Peptest achieved a pooled sensitivity of 0.62 (95% CI 0.49–0.73), a pooled specificity of 0.74 (95% CI 0.50–0.90), and an area under the curve of 0.70 (95% CI 0.66–0.74), which was consistent with previous findings, indicating that Peptest has a moderate diagnostic value for GERD/LPRD.

Because there are heterogeneity among studies, we did a subgroup and meta-regression analysis in this meta-analysis. First, there was no significant difference in diagnostic value between Asia and Western countries. There were 4 articles from Asia (2 from China, 1 from Japan, and 1 from Turkey), and 12 articles from Western countries (7 from the UK, 4 from Spain, Czech, Australia, and the USA).^[19,22,28–41]^

Second, the sample size (≥50 or <50) did not affect the diagnostic value. The sensitivity of Peptest for GERD/LPRD diagnosis varied from 33.3% to 85.0%, and the specificity varied from 25% to 100%. The study with the largest sample size was done by Wang et al, wherein 709 patients and 323 healthy controls were included, with an overall sensitivity of 85.0% and specificity of 60.0%.^[33]^ The diagnostic criteria of the studies with the largest and the second largest sample size (985 patients and 22 healthy controls) were questionnaires other than reflux monitoring, which might influence the diagnostic accuracy.^[30]^

Third, there was no significant difference in diagnostic value between the cut-off value of above 25 ng/ml and no more than 25 ng/ml. Usually, patients are instructed to clear saliva from the back of their throat by coughing and spit it into the collection tube containing 0.5 ml buffer (0.5 ml of 0.01 M citric acid) to prevent pepsin auto-digestion. After collection of salivary samples, saliva was stored at 4° (recommended) before measurement of pepsin by Peptest following a standard procedure. Briefly, 80 μl of sample from supernatant layer is collected after centrifugation and then mixed with 240 μl migration buffer. 80 μl of mixed sample is applied to the well of the Peptest lateral flow device (LFD), and the result can be read in the window of the LFD 15 minutes later.^[30]^ There are 2 lines in the LFD window. When the saliva sample is applied, the internal control line will be visualized, and t-line (for sample) will only be visualized when the pepsin is detected by LFD. A higher concentration of pepsin is reflected by a more intense t-line. By manual interpretation of the results, semiquantitative assessment of pepsin in the samples is carried out: samples with a pepsin concentration <16 ng/ml or 25 ng/ml is recognized as negative; concentration from 16 ng/ml to 75 ng/ml or 100 ng/ml was considered as weak positive; concentration from 100 ng/ml to 250 ng/ml was considered as moderate positive; and concentration above 250 ng/ml was considered as strong positive.^[22,33]^ Quantification of concentration of pepsin by Peptest can be obtained by a LFD reader (RD Biomed, Hull). Because the LFD reader is not available in all country, only 1 study has demonstrated a quantitative result of pepsin concentration.^[39]^ Although the t-line of Peptest will be visualized at the concentration above 16 ng/ml or 25 ng/ml, some researchers thought that samples with a pepsin concentration above 16 ng/ml or 25 ng/ml could not differentiate LPR/GERD patients from controls, so they used a higher cut-off value, such as 76 ng/ml or 210 ng/ml.^[33,34,37]^ In theory, if the cut-off value elevated, the sensitivity should go down, with the specificity going up. In order to evaluate the diagnostic value of negative control (≤25 ng/mL), a subgroup analysis with cut-off value >25 ng/ml or ≤25 ng/ml was performed. However, the subgroup analysis showed that there was no significant difference in diagnostic value between cut-off value >25 ng/ml (weak positive) or ≤25 ng/ml (negative). This result coincided with the research performed by Bobin et al, in which they found that saliva pepsin concentration of LPR patients was not correlated with reflux episodes by measuring pepsin concentration during MII-pH monitoring.^[46]^

Fourth, some studies enrolled healthy controls, while other studies included symptomatic controls.^[19,22,28–41]^ If only healthy controls were involved, the case–control design could not be avoided, leading to bias in patient sampling.^[24]^ Therefore, theoretically, the diagnostic value should be better in healthy controls. The subgroup analysis showed a higher sensitivity for LPR/GERD patients vs healthy controls (0.76[0.62–0.89]) when compared with that for LPR/GERD patients vs symptomatic controls (0.52[0.37–0.66], *P* = .02).

Fifth, the subgroup analysis showed that the sensitivity and specificity were lower in pH/pH-MII group than group using questionnaires or sighs (0.51 vs 0.76, *P* = .02 and 0.65 vs 0.86, *P* = .30, separately). Ambulatory reflux monitoring can provide confirmatory evidence of reflux episode of GERD/LPR, and is regarded as the golden standard for GERD/LPR. In contrast, the questionnaires have only 70% sensitivity and 67% specificity for GERD.^[47]^ The pH-MII monitoring can detect both distal refluxes and proximal refluxes. Salivary pepsin will only be detectable when the reflux of gastric content reaches the mouth. The salivary pepsin will not be easily detected when the gastric content reaches the lower part of the esophagus (distal refluxes). Matsumura et al showed that higher reflux burden was correlated with higher salivary pepsin concentration.^[28]^ Since the laryngeal symptoms and signs may indicate proximal refluxes, the salivary pepsin may be easier to detect. As a result, the sensitivity is low in pH/pH-MII monitoring group.

Last but not least, there was no significant difference in sensitivity for Peptest to diagnose GERD or LPR (0.59[0.42–0.77] vs 0.64[0.45–0.82], *P* = .51), whereas the specificity was lower in GERD group (0.62[0.40–0.85] vs 0.89[0.78–1.00], *P* = .02). Since LPR patients have solid proximal retrograde events, which could bring pepsin to oral and laryngeal, while GERD patients could only present as with distal refluxes. However, some studies showed that though Peptest may help to diagnose patients who have conclusive evidence of reflux, the level of pepsin saliva concentration is not associated with the reflux episodes in proximal at the MII-pH monitoring.^[28,46]^ More studies should be done to find the relationship between the pepsin concentration, reflux episodes, and related symptoms in the future.

Some researchers postulated a connection between LPR and GERD. Both were caused by the reflux of gastric and duodenal content, and LPR was regarded as one of the extraesophageal complications of GERD. Park et al found that the mean intercellular space of LPR was significantly increased, which was similar to GERD, suggesting common pathogenesis between LPR and GERD.^[48,49]^ Moreover, both GERD and LPR use ambulatory reflux monitoring as the golden standards. In 24 hours esophageal pH-MII monitoring, a retrograde bolus transit that crossed all impedance rings and reached the hypopharynx was defined as an LPR event.^[50,51]^ In addition, both GERD and LPR patients were enrolled in some studies as there was no clear borderline between the 2 diseases. As a result, the studies of both GERD and LPR were involved in this meta-analysis.

Peptest is not only used in GERD/LPRD diagnosis, but also in predicting therapy responses and other diseases relating to extraesophageal reflux. A prospective individual single-cohort study showed that Peptest in saliva/sputum samples with strong positive results were significantly associated with a good PPI response, with 79.2% of positive predictive value.^[52]^ A pilot trial showed that salivary pepsin could be a marker for the success of treatment, and median pepsin value decreased from 206.3 to 76.0 ng/ml in patients was defined as laparoscopic antireflux surgery success.^[53]^ Iannella et al showed that a high number of patients with obstructive sleep apnea seemed to show positivity for salivary pepsin, and direct correlation between body mass index and the value of salivary Peptest was observed.^[54]^ In order to confirm extraesophageal reflux, Peptest was used to detect pepsin in middle ear fluid obtained during myringotomy in children with chronic otitis media with effusion.^[55]^

This article is the most comprehensive meta-analysis estimating the diagnostic value of salivary Peptest for GERD/LPRD up to date, with 16 articles included. Moreover, it is the first meta-analysis focused on Peptest. The first systemic review of using pepsin as a diagnostic tool for LPR was done by Christian et al, although all kinds of methods for pepsin detection were included, there was no pooled sensitivity and specificity in the study.^[42]^ Wang et al did the first meta-analysis of salivary pepsin for LPR. The study encompassed 11 studies described a moderate value of pepsin determination in saliva for the diagnosis of LPR, with a pooled sensitivity of 64% (95% CI 0.43–0.80) and specificity of 68% (95% CI 0.55–0.78), which was similar to our results. However, that meta-analysis included all kinds of methodology for pepsin detection, such as Peptest, western blot, enzyme-linked immunosorbent assay and fibrinogen digestion.^[43]^ Although only LPR was mentioned in the title and abstract of the 2 articles mentioned above, they included studies of both GERD and LPR.^[42,43]^ Guo et al did a meta-analysis of salivary pepsin for GERD diagnosis, with only 5 articles included.^[45]^ Zhang et al did a systematic review and network meta-analysis to assess different diagnostic tests for gastroesophageal reflux disease, only 2 studies of Peptest were included.^[44]^ Nine studies without using Peptest were excluded in our study, which included:

(1) Two studies^[18,21]^ using fibrinogen digestion method (failure to obtain diagnostic value for GERD/LPR);
(2) Three studies using western blotting (Kim et al enrolled 40 patients with clinically suspected manifestations of GERD and showed sensitivity of 89% and specificity of 68% based on 24-hour pH-metry data.^[20]^ The other 2 studies failed to obtain diagnostic value for GERD/LPR^[57,58]^), and
(3) Four studies using enzyme-linked immunosorbent assay (1 is for saliva as a possible risk factor for early laryngeal cancer,^[59]^ other 3 studies failed to obtain diagnostic value for GERD/LPR^[60–62]^).

The limitations of this study should be mentioned. First of all, although 16 studies were enrolled, the sample size was relatively small, with 2401 patients and 897 controls included. Additionally, there were no standard protocols for Peptest, such as the best time and frequency of saliva sampling, which might result in inconsistence of diagnostic value. Na et al reported that the concentration of pepsin in saliva collected upon waking was significantly higher than that collected at any other time in patients with LPR symptoms and solid evidence of proximal esophageal reflux.^[56]^ In contrast, Hayat et al reported that GERD patients had more often postprandial positive samples and suggested the necessity for multiple postprandial sampling during the 24 hours periods.^[39]^ Matsumura et al showed that the most suitable time for collection was 1 hour after evening meals.^[28]^ The different protocols, study design, patient cohort, and cut-off value might lead to heterogeneity. Finally, the summarized data in some articles constrained us from conducting a more detailed analysis.

## 5 Conclusion

The meta-analysis showed that Peptest in saliva is with moderate diagnostic value for LPR/GERD. What's more, further large-scale studies with standard protocols should be made to verify the results.

## Author contributions

**Conceptualization:** Zihao Guo, Chuan Zhang, Jiali Jiang, Hao Wu, JinXia Zhu, Shutian Zhang.

**Data curation:** Zihao Guo, Chuan Zhang, Jiali Jiang, Hao Wu, Shutian Zhang.

**Formal analysis:** Zihao Guo, Hao Wu.

**Funding acquisition:** Shutian Zhang, Chuan Zhang.

**Investigation:** Zihao Guo, Chuan Zhang, Jiali Jiang, Hao Wu.

**Methodology:** Zihao Guo, Hao Wu.

**Project administration:** Jiali Jiang, Hao Wu, JinXIa Zhu, Shutian Zhang, Chuan Zhang.

**Resources:** Jiali Jiang.

**Software:** Zihao Guo, Chuan Zhang, Jiali Jiang, Hao Wu.

**Supervision:** Chuan Zhang, JinXia Zhu, Shutian Zhang.

**Validation:** Jiali Jiang.

**Writing – original draft:** Zihao Guo.

**Writing – review & editing:** Zihao Guo, Shutian Zhang, Chuan Zhang.
